# Prospective analysis of Kawasaki disease cases in Catalonia (Spain) from March 2013 to March 2014

**DOI:** 10.1186/1546-0096-12-S1-P349

**Published:** 2014-09-17

**Authors:** Judith Sanchez-Manubens, Jordi Anton, Fredy Prada, Estibaliz Iglesias, Joan Calzada-Hernandez, Samuel Hernandez, Vicenç Torrente-Segarra, Silvia Ricart, Sergi Borlan, Clara Gimenez Roca, Marc Tobeña, Socorro Uriz, Anna Fernandez, Maria Mendez, Alvaro Diaz, Olga Calavia

**Affiliations:** 1 Rheumatology Unit, Department of Pediatrics, Hospital Sant Joan de Déu, Esplugues de Llobregat, Spain; 2 Rheumatology Unit, Department of Pediatrics, Hospital Parc Taulí, Sabadell, Spain; 3 Cardiology Department, Hospital Sant Joan de Déu, Esplugues de Llobregat, Spain; 4 Pediatrics Department, Hospital Vall d'Hebron, Barcelona, Spain; 5 Pediatrics Department, Consorci Sanitari de Terrassa, Terrassa, Spain; 6 Pediatrics Department, Hospital Arnau de VIlanova, Lleida, Spain; 7 Pediatrics Department, Hospital Germans Tiras i Pujol, Badalona, Spain; 8 Pediatrics Department, Hospital de Nens, Barcelona, Spain; 9 Pediatrics Department, Hospital Joan XIII, Tarragona, Spain

## Introduction

Kawasaki disease (KD) is an acute self-limited systemic vasculitis relatively common in childhood. In Japan, last published survey shows an incidence up to 239.6/10^5^ children <5 years old (yo). In Madrid (Spain) a retrospective study with no well defined reference area showed an incidence of 15.1/10^5^ children <5yo.

## Objectives

To ascertain the incidence and clinical features of KD in Catalonia Catalonia, autonomous region in northeast Spain with 7.5 million inhabitants, over a prospective period of one year.

## Methods

Observational population-based study including all Catalan hospitals with Pediatric Units, both public and private management. Prospective communication of new cases of KD was performed from March 2013 to March 2014. The presence of coronary aneurysms (CA) in echocardiology was based in the body surface area according to the American Heart Association. Giant CA was considered if the CA was higher than 8mm.

## Results

The study included 33 different hospitals from Catalonia. Over the one-year study period 49 new cases of KD were collected. The annual incidence was 4.1/10^5^ children <14yo and 12.3/10^5^ children <5yo (mean age 34±23months (m), range 3.3-105.1m). There were not differences between boys and girls. Mean delay between onset of the disease and diagnostic was 8.6±9.8 days. Ethnic distribution was: Caucasian 42 patients (85.7%), North African 4 (8.1%), Amerindian 2 (4%) and Asian 1 (2%). Distribution of classical manifestations for KD was: fever in 100% of patients, changes in extremities: edema and erythema 51%% and desquamation 44.9%, exanthema 85.7%, conjunctival injection 91.8%, changes in lips and oral cavity 77.5% and lymphadenopathy 24.8%. Other clinical findings reported were: sterile pyuria in 10 (20%) patients, nausea and vomiting in 13 (26.5%), abdominal pain in 12 (24.4%), gallbladder distention in 1 (2%), transaminase elevation in 13 (26.5%), jaundice in 3 (6.1%), irritability in 20 (40.8%), and arthritis or arthralgia in 13 (26.5%). Cardiologic findings were: perivascular brightness of the coronary wall in 8 (16.3%) patients, myocarditis in 1 (2%), mitral regurgitation in 3 (6%) and CA in 8 (16.3%) patients, disappearing before the 2^nd^ moth of disease in 2 patients. No gyant CA were reported. Intravenous immunoglobulin (IVIG) was administered in 47 (95.9%) patients with response to the 1^st^ dose in 40 (91.6%). Day of IVIG administration was 7.8±3.3. Abciximab was administered in 2 patients. 97.9% of patients received anti-platelet dose aspirin in the convalescent phase.

## Conclusion

During the prospective period incidence of KD in Catalonia (Spain) was higher than the one ascertained in the retrospective analysis. Further analysis may be performed in order to know if this is due to a better diagnosis, a better registry o a real incidence increase. It seems to be a higher incidence of CA in our cohort despite high rates of treatment response. Further analysis is required. Incidence rate, other clinical features and treatment plans are similar to dose described in studies in other European countries.

## Disclosure of interest

None declared

